# Supplementation of Micro- and Macronutrients—A Role of Nutritional Status in Non-Alcoholic Fatty Liver Disease

**DOI:** 10.3390/ijms25094916

**Published:** 2024-04-30

**Authors:** Magdalena Tyczyńska, Gabriela Hunek, Martyna Szczasny, Adam Brachet, Jacek Januszewski, Alicja Forma, Piero Portincasa, Jolanta Flieger, Jacek Baj

**Affiliations:** 1 Department of Correct, Clinical and Imaging Anatomy, Medical University of Lublin, Jaczewskiego 4, 20-090 Lublin, Poland; m.tyczynska@onet.pl; 2 Chair and Department of Forensic Medicine, Medical University of Lublin, Jaczewskiego 8b, 20-090 Lublin, Poland; hunekgabriela@gmail.com (G.H.); adambrachet@gmail.com (A.B.); 3 Chair and Department of Anatomy, Medical University of Lublin, Jaczewskiego 4, 20-090 Lublin, Poland; martynaszczasny@gmail.com (M.S.); jacek.januszewski000@gmail.com (J.J.); 4 Clinica Medica “A. Murri”, Department of Biomedical Sciences & Human Oncology, University of Bari Medical School, 70124 Bari, Italy; piero.portincasa@uniba.it; 5 Department of Analytical Chemistry, Medical University of Lublin, Chodźki 4A, 20-093 Lublin, Poland; jolanta.flieger@umlub.pl

**Keywords:** non-alcoholic fatty liver disease, micronutrients, macronutrients, nutrition, vitamins, unsaturated fats, carotenoids, trace elements

## Abstract

Non-alcoholic fatty liver disease (NAFLD) is a condition in which the pathological cumulation of fat with coexisting inflammation and damage of hepatic cells leads to progressive dysfunctions of the liver. Except for the commonly well-known major causes of NAFLD such as obesity, dyslipidemia, insulin resistance, or diabetes, an unbalanced diet and imbalanced nutritional status should also be taken into consideration. In this narrative review, we summarized the current knowledge regarding the micro- and macronutrient status of patients suffering from NAFLD considering various diets and supplementation of chosen supplements. We aimed to summarize the knowledge indicating which nutritional impairments may be associated with the onset and progression of NAFLD at the same time evaluating the potential therapy targets that could facilitate the healing process. Except for the above-mentioned objectives, one of the most important aspects of this review was to highlight the possible strategies for taking care of NAFLD patients taking into account the challenges and opportunities associated with the micronutrient status of the patients. The current research indicates that a supplementation of chosen vitamins (e.g., vitamin A, B complex, C, or D) as well as chosen elements such as zinc may alleviate the symptoms of NAFLD. However, there is still a lack of sufficient data regarding healthy ranges of dosages; thus, further research is of high importance in this matter.

## 1. Introduction

Non-alcoholic fatty liver disease (NAFLD) has a global prevalence of 25% and is the main cause of cirrhosis and hepatocellular carcinoma (HCC) [1]. It is characterized as a continuous pathogenic process that begins with steatosis and with the possible coexistence of mild inflammation (non-alcoholic fatty liver) which ends with non-alcoholic steatohepatitis (NASH)—a necroinflammation and faster fibrosis progression [2]. Due to its high prevalence, NAFLD is becoming the fastest-growing cause of liver-related mortality worldwide and is now the most common origin of chronic liver disease worldwide. The occurrence varies from 13.5% in Africa to 31.8% in the Middle East, with the difference likely being the result of caloric intake, physical activity, fat tissue distribution, socioeconomic status, and genetics [3]. Moreover, due to its close association with metabolic syndrome, NAFLD occurs in 47.3–63.7% of people with type 2 diabetes (T2DM) and about 80% of people with obesity. The illness possesses a genetic component, which can increase the disease risk by 20–70% [4]. Nucleotide polymorphism in the patatin-like phospholipase domain-containing protein 3 (*PNPLA3*) gene, is the best-known genetic variation linked to the proneness of NAFLD [5]. The biggest issue, as well as the main cause of NAFLD, is excessive nutrition, which in consequence expands adipose depots and leads to the accumulation of ectopic fat. This imbalance leads to the formation of lipotoxic lipids that promote cellular stress, and activate inflammasome and cell apoptosis, followed by stimulation of inflammation, tissue regeneration, and fibrogenesis [6,7]. In addition, mitochondria, with their significant role in lipid metabolism, reactive oxygen species (ROS), and ATP production, as well as apoptosis, have been associated with the progression of simple steatosis to NASH as well as the progression of NAFLD [8]. In fact, it is primarily a high-fat diet that ultimately leads to the impairments of lipid metabolism, induction of apoptosis, and production of ROS which leads to the onset of NAFLD. A high-fat diet may result in increased endoplasmic reticulum stress, impaired autophagy, or mediation of various inflammatory responses [9]. Furthermore, studies indicate that mitochondrial dysfunctions induced by a high-fat diet may also play a crucial role in the NAFLD progression via such processes as the stimulation of ROS production by the accumulated damaged mitochondria [10]. The data suggest that an improved diet quality and sustained or increased exercise routine reduce the risk of developing NAFLD, even among individuals with high genetic risk [11]. Additionally, we recognize drugs that may improve NASH histology, such as vitamin E, obeticholic acid, and pioglitazone [12]. Obesity is a well-known NAFLD risk-increasing factor and, in particular, visceral obesity is a well-known and significant risk factor for complications of metabolic syndrome. Given that information, the assessment of waist circumference may be more accurate in overall disease risk valuation [13]. Another important risk factor is the existence of T2DM. Patients with said condition present a higher frequency of NAFLD (40–70%) and are more likely to develop advanced fibrosis, cirrhosis, and HCC than people without diabetes [14]. Another related variable is age; when it increases, so does the prevalence of NAFLD and NAFLD-related fibrosis. These prevalence rates may be driven by a higher frequency of metabolic conditions in older individuals. Other studies confirmed these results linking age to an increased risk of severe hepatic fibrosis, HCC, and T2DM [15,16]. Moreover, reports from Sri Lanka and Thailand found the prevalence of NAFLD to be 4.2% higher in women. On the contrary, several studies from the US, Southwest China, and Spain have described a higher prevalence of NAFLD in men [17,18]. The most common risk factors of NAFLD include high triglyceride and low HDL-cholesterol levels, metabolic syndrome with an emphasis on obesity, or type 2 diabetes. However, since the liver plays a crucial role in micronutrient metabolism, in the following narrative review we aimed to analyze the impact and relationship of micro- and macronutrient status on the possible onset and progression of NAFLD.

## 2. Role of Trace Elements in Hepatic Inflammation

Hepatic inflammation is activated with the proinflammatory cytokines and chemokines produced by adipocytes, hepatic macrophages, and lipid-laden hepatocytes. The above-mentioned factors then promote the activation of stellate cells, which are responsible for fibrogenesis in the liver [19].

### 2.1. Manganese

Manganese (Mn) is an important hepatic trace element for several enzymes, that for instance catalyze the last step in the hepatic urea cycle [20]. In animals, the lack of Mn can impair insulin production, alternate lipoprotein metabolism, damage the oxidant defense system, and perturbate the metabolism of growth factors [21]. Rodents, treated orally with high doses of Mn salts, showed no retention of excess concentrations in any tissue, suggesting that the rate of accumulation and overall toxicity of Mn is relatively low. Confirmed cases of human Mn toxicosis are currently restricted to high levels of airborne Mn exposure patients and cases with Mn excretory pathways disruption [22]. 

### 2.2. Aluminum

Aluminum (Al) is one of the most widely used, non-toxic metal in daily life. Many factors may cause unwanted high intake of Al, such as medical exposure, excessive dietary intake, and contaminated drinking water. Al properties disturb Fe homeostasis, biological membranes, enhance ROS formation, and damage DNA, which promotes mitochondrial dysfunction and intracellular lipid accumulation in hepatocytes [23]. Hepatic cell exposure creates a hypoxic environment, promotes oxidative stress (OS), and stimulates anaerobic metabolism. Excessive uptake of Al salts may impact Fe deposition and expression of transferrin in adult mice liver [24]. Due to biliary excretion being a significant pathway for Al systematic exertion, patients diagnosed with liver disease are more predisposed to Al accumulation [25].

### 2.3. Cadmium

Cadmium (Cd) is a toxic transition metal with carcinogenic potential. This heavy metal accumulates primarily in the liver and kidney where, like Zinc, it binds to metallothioneins (MTs). Therefore, when there is a shortage of MT or modifications in Zn metabolism, the normally tolerated intracellular Cd concentration may become toxic [26]. People occupationally exposed to Cd, by working the in metal or battery-producing industry, may be at risk for Cd poisoning. The symptoms of Cd poisoning include softening of the bones and kidney failure [27]. Massive tobacco consumption may be another source of elevated Cd intake [28]. Acute overload may then lead to elevated ROS levels and tissue damage [29]. On the contrary, it has been observed that chronic Cd exposure triggers oncogene overexpression and alters the activity of critical transcription factors such as AP-1 and NF-κB that are linked to tumorigenesis and malignant cell transformation [30].

### 2.4. Chromium

Chromium (Cr) III occurs in trace amounts in foods and water and has genotoxic and mutagenic potential [31]. Based on historical reports, it was supposed that Cr increases the action of insulin and that a special chemical form, termed Glucose Tolerance Factor, is a suitable oral medication for diabetes [32]. Contrary to this assumption, a recent meta-analysis indicates that, in patients with T2DM, Cr supplementation has no significant effect on body mass index, lipid profiles, and glycosylated hemoglobin [33]. Risk analysis in a Chinese cohort study showed that occupational Cr poisoning led to an increased risk of lung or liver cancer in male workers [34]. 

### 2.5. Lead

Lead (Pb) and its compounds can be absorbed through food, by inhalation, or through the skin. Inorganic Pb poisoning damages the central and the peripheral nervous system. It has an effect on blood formation, gastrointestinal disorders induction, and kidney damage. Absorbed or inhaled inorganic Pb is not metabolized and therefore it excretes unchanged, mainly in the urine [35]. Furthermore, it has been said that, in male soft tissues, Pb concentrations are up to 30% higher than in equivalent female tissues [36]. Organic, environmental Pb undergoes oxidative dealkylation within the liver by a cytochrome P450-dependent monooxygenase reaction, and even at non-toxic concentrations, it was observed to aggravate OS toxicity [37].

## 3. Micronutrients Involved in NAFLD

### 3.1. Iron

Iron is obtained from dietary sources that consist of heme (≤10%) and non-heme (>90%) compounds. Intestinal absorption is the main process by which the body obtains iron, with enterocytes in the duodenum primarily responsible for absorbing 1–2 mg of iron daily [38,39].

Dysmetabolic iron overload syndrome (DIOS) is a recently acknowledged condition found in approximately one-third of patients diagnosed with both NAFLD and metabolic syndrome [40]. DIOS is a medical condition characterized by a minor elevation in liver and systemic iron levels, for which the underlying cause of the surplus iron remains unclear [41]. This condition is characterized by high serum ferritin levels, moderately elevated transferrin saturation, and symptoms of metabolic syndrome, including diabetes, hypertension, and hypercholesterolemia [42]. A study investigating middle-aged men with unexplained mild-to-moderate liver iron accumulation found a consistent association with insulin resistance [43]. The precise mechanisms by which iron affects insulin signaling remain uncertain, but a recent study discovered that β-cells in the pancreas express hepcidin, indicating a potential involvement in regulating both glucose and iron levels within the body [44]. Iron accumulation in β-cells can result in oxidative damage due to their heightened susceptibility to prolonged OS, which has been demonstrated to impede insulin secretion from the pancreas [45]. Iron-induced OS can lead to inflammation by activating TNF-α, which in turn inhibits insulin signaling by decreasing the expression of glucose transporter 4 (GLUT4) and the phosphorylation of insulin receptor substrate 1 (IRS-1) [46]. 

The two-hit hypothesis is a prominent theory explaining the development of NASH from the fatty liver [47]. According to this theory, visceral fat-induced insulin resistance is the primary hit. Consequently, the elevated presence of fatty acids in the bloodstream leads to hepatic fat accumulation. This impact enhances the liver’s susceptibility to various factors that contribute to subsequent hits and facilitates liver injury [48]. The second “hit” is triggered by multiple stressors that result in strain and inflammation of the liver. Hepatic inflammation leads to cytokine production, which continues the recruitment of inflammatory cells and causes tissue deterioration [49]. Iron catalyzes the generation of ROS, leading to the hypothesis that the deposition of iron in the liver serves as the second hit. Recent research indicates that iron may play a role in additional physiological processes, such as altered lipid metabolism and insulin signaling. This leads to the belief that iron is also involved in the early development of steatosis and the progression of NASH [50]. OS and cytokine damage can lead to cell death, triggering the subsequent release of cytokines by phagocytic macrophages. This process subsequently induces the activation of hepatic stellate cells, resulting in the initiation of fibrogenesis [42,51,52,53]. 

George et al. [54] conducted a study that proposed a correlation between increased stainable hepatic iron or biochemical hepatic iron content (HIC) and the presence of more advanced fibrosis in patients with NAFLD. Subsequent research has yielded conflicting results. There is debate regarding the association between hepatic iron levels and the progression of NASH to an advanced stage. Some studies have reported a connection [55,56], whereas others have not observed any such relationship [51,57,58,59,60]. The etiology of hepatic iron accumulation in liver diseases, such as NAFLD, remains poorly identified. Hepatic iron distribution can manifest in three distinct patterns: exclusive localization within hepatocellular cells (HCs), exclusive localization within reticuloendothelial system (RES) cells, or a combination of both HC and RES cells [61,62]. In Nelson et al.’s study [63], hepatic iron staining was observed in 34.5% (293 out of 849) of patients exhibiting any of the above-mentioned histological patterns. Patients exhibiting the RES iron-staining pattern demonstrated a greater propensity for advanced fibrosis in comparison to individuals with HC iron. Moreover, patients who had RES iron exhibited a greater occurrence of advanced histological characteristics such as fibrosis, portal inflammation, hepatocellular ballooning, and definitive nonalcoholic steatohepatitis when compared to patients with hepatocellular or mixed iron patterns. After adjusting for age, gender, diabetes status, and BMI, the study revealed that the presence of RES iron was independently linked to advanced hepatic fibrosis in the multiple regression analysis. Iron deposits related to RES iron were found to be associated with more severe disease, characterized by advanced histologic features, a higher mean NAFLD Activity Score (NAS), and clinical indicators such as elevated serum aminotransferases and decreased platelets. However, it should be noted that HC iron deposits exhibited less severe histologic and clinical characteristics in comparison to the other patterns. The mixed iron group exhibited intermediate disease severity. 

Studies indicate that hepcidin may play a role in the development of the disease. Research has indicated that individuals with NAFLD, particularly those in advanced stages like NASH, exhibit increased levels of hepcidin [64]. Elevated hepcidin levels in the liver are associated with the accumulation of iron in liver macrophages, particularly Kupffer cells [65]. New research suggests that iron can exacerbate and accelerate NAFLD by activating the redox-sensitive transcription factor NF-kB in Kupffer cells. This activation leads to the initiation of inflammatory, fibrosis-promoting, and cytotoxic pathways [66,67,68]. It is postulated that the production of ROS is correlated with the histopathological manifestations of iron excess in the liver. ROS initiates a series of events that result in OS, causing lipid peroxidation, mitochondrial dysfunction, endoplasmic reticulum (ER) stress, inflammation leading to tissue necrosis, and apoptosis [69]. ROS have been observed to possess both direct cytotoxic properties and the ability to stimulate the up-regulation of NF-κB, a protein implicated in the activation of inflammatory, fibrogenic, and apoptotic pathways [66,67,68,70,71]. Iron-induced reactive oxygen and nitrogen species interact with the small G-protein p21ras, activating TGF-activated kinase-1 (TAK1), phosphoinositide-3 kinase (PI3K), and mitogen-activated protein kinase-1 (MEK1), all of which are known NFκ-B regulators [72]. NF-κB subsequently induces the upregulation of the cytokines TNF-α and IL-6, which play pivotal roles in hepatic inflammatory and fibrogenic reactions [73,74]. The activation of hepatic stellate cells (HSCs) is a crucial step in the paracrine signaling cascade of fibrogenesis. This activation triggers morphological alterations in dormant cells, leading to their differentiation into proliferative myofibroblasts. Myofibroblasts are crucial in synthesizing essential constituents of the extracellular matrix, such as collagen types I and III [75]. 

**Figure 1:**
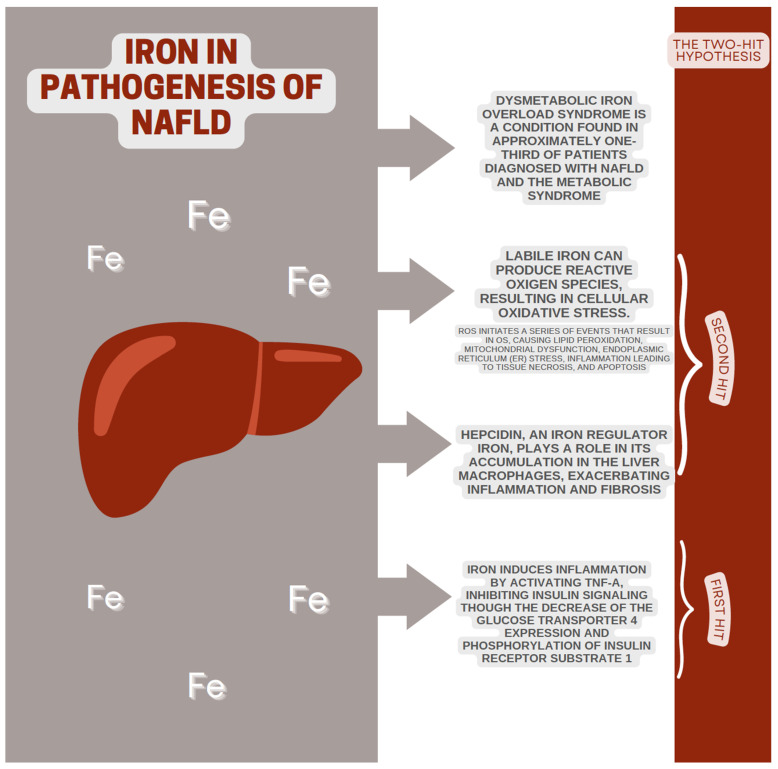
Scheme for the role of iron in the pathogenesis of NAFLD (this figure was created in Canva version 2.261.0).

A meta-analysis [76] revealed no significant disparity in dietary iron intake between individuals with NAFLD and control groups. The disparity became notable primarily in Asia, where the intake of iron was higher among the group of individuals without health issues. This discovery holds significant significance, as a study conducted on the Chinese population [77] revealed a positive correlation between dietary iron intake and the likelihood of developing NAFLD, indicating a dose–response relationship. Nevertheless, this correlation seems to be evident among males but not among females. The combination of this information suggests that iron absorption is dysregulated in individuals with NAFLD (Figure 1).

### 3.2. Copper

The overall copper concentration within the human adult body ranges from 50 to 120 mg. The presence of an adequate quantity of copper is essential for the maintenance of optimal biological functioning [78], as it plays a vital role in lipid metabolism, mitochondrial activity, iron metabolism, and antioxidant defense mechanisms. 

Dysregulation of copper levels may play a role in the development of NAFLD [79]. Copper is a vital micronutrient that plays a crucial role as a structural and enzymatic cofactor in several antioxidant proteins, including cytochrome c oxidase (COX), superoxide dismutase (SOD), and ceruloplasmin [80]. According to the research, the reduction in hepatic ceruloplasmin leads to the replenishment of hepatic copper levels, hence mitigating liver steatosis through the modulation of the copper-SOC1-AMPK signaling pathway in a mouse model of NAFLD [78]. Previous studies have demonstrated that a deficiency in dietary copper can contribute to the development of steatosis and insulin resistance in Sprague Dawley rats [81]. Furthermore, it has been observed that individuals diagnosed with NAFLD have a 50% reduction in hepatic copper levels when compared to both control groups and individuals with liver impairment unrelated to NAFLD [81]. Consistent with these findings, there was an observed negative correlation between hepatic copper levels and the severity of steatosis in obese individuals. Furthermore, it was shown that patients with little or mild hepatic steatosis had greater levels of hepatic copper content compared to lean participants [82]. The rats who were provided with a diet lacking in copper and rich in fructose for 4 weeks exhibited a greater degree of apoptosis and steatosis compared to the rats that were given a diet containing adequate levels of copper [83]. It has also been seen that rats subjected to a diet low in copper would spontaneously develop hepatic steatosis [84]. Additionally, individuals with NAFLD who also exhibit hepatic copper deficiency tend to experience more pronounced liver steatosis, inflammation, and clinical symptoms [81,85]. 

Another method by which copper deficiency contributes to the development of NAFLD is its interaction with iron. A shortage in copper has the potential to decrease the expression of ferroportin-1 and the activity of ceruloplasmin ferroxidase, thus impeding the export of iron from the liver and leading to the accumulation of iron in this organ [86,87,88]. According to reports, there is an association between reduced hepatic copper levels and NAFLD. However, it should be noted that excessive copper can also have detrimental effects on hepatocytes [89,90]. This is because excessive copper has the potential to contribute to the formation of ROS, which can impair liver function [88]. The discovery of the efficacy of many antioxidant chemicals in combating NAFLD through copper binding is a noteworthy observation. This underscores their potential importance in impeding the advancement of the disease. The substances include curcumin, epigallocatechin-3-gallate (EGCG), luteolin, luteolin-7-glucoside, caffeic acid, caffeine, oleuropein, quercetin, rutin, and resveratrol [91]. 

In their study, Lan et al. [92] discovered that elevated copper levels exhibited a significant protective influence against NAFLD, specifically in males, but no such benefit was observed in females. Chen et al. [93] demonstrate that there is a significant elevation in serum copper levels in individuals diagnosed with NAFLD when compared to those without the condition. Specifically, their findings reveal that, after adjusting for various confounding factors, the risk of NAFLD was shown to increase by 97% in those belonging to the highest quartile of copper levels in comparison to those in the lowest quartile. The findings from a meta-analysis [94] comprising six articles and involving a total of 2607 patients with NAFLD and 1441 non-NAFLD controls revealed a significant decrease in hepatic copper concentration among NAFLD patients ([SMD] = −0.98, 95% [CI] = [−1.21; −0.74], *p* < 0.0001). 

While there is a correlation between obesity and a higher likelihood of developing NAFLD, it is crucial to acknowledge that not all individuals conform to this particular phenotype. Nunes et al. [95] identified a unique phenotype among individuals diagnosed with NAFLD characterized by reduced ceruloplasmin levels (below 25 mg/dL). They were found to exhibit a lower BMI, decreased levels of low-density lipoprotein (LDL), HDL, and total cholesterol, as well as increased levels of ferritin, in comparison to individuals with lower ceruloplasmin levels. The study conducted by Porcu et al. [96] showed a positive association between elevated copper serum levels and the occurrence, malignant advancement, and recurrence of many types of human cancers, including HCC. The data also suggest a positive correlation between elevated levels of circulating copper and the progression of NAFLD-cirrhosis toward HCC. This indicates that copper could potentially serve as a novel biomarker for hepatocyte transformation. Specifically, a cut-off value of 163.3 μg/dL for copper levels can be utilized to distinguish NAFLD-cirrhotic patients who are more likely to develop HCC.

### 3.3. Zinc

Zinc is an essential trace mineral for liver lipid metabolism. It stimulates hepatic cell lipophagy, reducing lipid accumulation and increasing lipolysis. It regulates enzyme production, cell signaling, antioxidant defense, insulin biosynthesis and secretion, and mRNA expression [97,98,99]. According to estimates by the WHO, zinc deficiency affects about a third of the world’s population [100]. When compared with controls, NAFLD patients had significantly lower serum zinc levels [101]. Decreased zinc levels were linked to progressive hepatic fibrosis in biopsy-diagnosed NAFLD patients [102]. A cross-sectional study analyzed 300 NAFLD patients using the NAFLD liver fat score [103]. The fibrosis-4 index [104] showed a link between higher stages of hepatic fibrosis and zinc deficiency. The liver maintains systemic zinc homeostasis in the body [105]; thus, chronic liver diseases can lead to zinc deficiency. 

Numerous studies have examined how a high zinc intake reduces liver damage and prevents hepatic steatosis. Both in vivo and in vitro studies show that zinc supplementation reduces liver and hepatocyte lipid deposition, attenuating NAFLD. It also significantly reduced serum AST and ALT, which are important measures to evaluate liver injury [106]. IL-6 protein expression and MDA levels in HepG2 cells decreased with zinc administration. This study shows that zinc reduces lipid peroxidation and liver damage. Patients with NAFLD may develop zinc insufficiency due to several reasons. OS, inflammation, insulin resistance, diabetes, obesity, dyslipidemia, and impaired gastrointestinal zinc absorption raise zinc supplementation needs [107]. Interestingly, high blood zinc levels are associated with NAFLD risk in one study [108]. This study proposes a hypothesis according to which zinc utilization and transport issues may develop from liver injury in early NAFLD, raising Zn levels to normal. Antioxidant stress and lipid metabolism enhance zinc consumption, causing a zinc shortage in severe NAFLD. 

The occurrence of ER stress leads to the activation of an unfolded protein response (UPR), which is a cellular adaptive mechanism aimed at mitigating the demand for protein folding. Hepatocytes’ extended UPR causes steatosis, while end-stage and resistant UPR causes steatohepatitis and HCC [109]. ER zinc levels must be sufficient to adjust to OS and minimize inflammation. Zinc supplementation reduces oxidative damage and regulates inflammatory cytokines like TNF-alpha [110,111,112,113]. Zn deficiency originates from increased demand for ER stress, but zinc deficiency itself can induce ER stress [114]. Animal studies showed that zinc and zip14 reduce apoptosis, steatosis, and regulate ER stress [115]. Zn deficiency causes the inactivity of SOD, which has strong antioxidant activity, which results in the overproduction of ROS. Notably, lower SOD and higher ROS states induced by Zn deficiency can lead to DNA damage, protein modification, and carcinogenesis [116]. Treatment with a nano-formulation of luteolin with Zn oxide in the form of Lut/ZnO NPs improved the antioxidant system, liver function, and OS markers [117]. 

Zinc is essential for insulin production, storage, secretion, and signaling [118]. Pancreatic beta cells store zinc-insulin crystals [119]. In diabetic patients, Zn content in the pancreas is 50% lower, with multiple studies demonstrating that people with type 1 or 2 diabetes have lower serum zinc levels than those without diabetes [109,119,120,121]. An ultrasound-diagnosed cross-sectional study of 2839 type 2 diabetic patients (T2DM) indicated that 70% had NAFLD [122]. Diabetes mellitus can cause zinc insufficiency due to zinc transporter dysfunction [119,123]. Low zinc absorption, glycosuria, and hyperglycemia can cause hyperzincuria [124,125,126,127], which has been reported by Kinlaw et al. [126], showing a positive correlation between urinary zinc excretion and serum glucose levels in patients with T2DM. Enhanced OS and hyperinsulinemia can also cause a zinc shortage due to an increased demand for that element, which results in an intracellular depletion of Zn [128,129,130]. Hyperinsulinemia diminishes pancreatic zinc reserves, which increases insulin production and lowers zinc levels [129]. A novel treatment is Lut/ZnO, which can manage NAFLD by improving insulin resistance, a significant NAFLD cause [117]. Ahmed et al. [117] found that Lut/ZnO NPs activate the PI3K/AKT signaling pathway, inhibiting FoxO1 and enhancing hepatic cell insulin sensitivity. In a study of 191 patients with NAFLD, Zn levels were negatively correlated with HOMA-IR (r = −0.284, *p* < 0.001) and NAFLD fibrosis score (r = −0.261, *p* < 0.001). The study found that lower serum zinc levels promote liver fibrosis but not inflammation [102]. Zinc intake improves glucose tolerance in advanced liver cirrhosis patients [131,132] and reduces hyperglycemia and hyperinsulinemia in db/db mice [133]. However, several investigations have identified no correlation between serum zinc levels and hepatic steatosis, steatohepatitis, and fibrosis, i.e., in bariatric surgery patients with NAFLD [134], or a retrospective cross-sectional investigation that found no significant variation in serum zinc levels between steatosis, NASH, and fibrosis grades [135]. Interestingly, mice fed a high-fat diet (HFD) for 12 weeks had reduced glucose clearance and body composition alterations, as expected, but Zn supplementation for eight weeks after the HFD did not affect glucose management, plasma transaminases, steatosis, or hepatic gene expression. This study found that 8 weeks of zinc supplementation cannot reverse established NAFLD. HFDs may have caused NAFLD to proceed to a stage wherein zinc treatment for 8 weeks cannot significantly improve [136]. In contrast to Qi et al. [106], which provided Zn supplementation alongside an HFD for 14 weeks, the cited study added it in the last 8 weeks following disease progression. This hypothesis suggests that, to attain therapeutic outcomes, the administration of supplements may be necessary far before the onset of NAFLD to obtain the intended reparative advantages.

Zinc’s interaction with leptin may regulate appetite and prevent obesity [137]. Zinc is linked to fat accumulation and glucose metabolism, and according to Olechnowicz et al. [138], it also regulates fat metabolism via the sterol regulatory element-binding protein (SREBP) family [139]. Patients with obesity had considerably lower serum zinc levels than those without obesity [140]. According to Fathi et al. [141], overweight/obese NAFLD patients showed a decrease in anthropometric parameters after 12 weeks of zinc supplementation and a calorie-restricted diet (*p* < 0.001). In an animal study utilizing rats, zinc and selenium co-supplementation after disease development was shown to reverse the progression by improving serum biochemical parameters (e.g., lipid profile and liver enzyme tests) and fat granule accumulation and size in the liver [142]. Long-term, dose-dependent studies using more specific markers are needed to clarify how zinc impacts obesity in NAFLD patients.

Wei et al. [97] found that Zn supplementation reduces lipid content in hepatocytes by activating fatty acid oxidation and blocking lipogenesis, while Qi et al. [106] proposed that Zn decreases aerobic oxidation and increases glycolysis, indicating that zinc changes cellular metabolism. The latter study theorizes that ROS generation during aerobic oxidation may cause OS, which frequently accompanies obesity. A study examined lipogenesis with Lut/ZnO NPs and showed that the compound downregulated SREBP1c to limit lipogenesis, lowering triacylglycerol and total cholesterol and raising HDL cholesterol [117]. A different study using zinc oxide nanoparticles (ZnO NPs) showed that, by holding SREBP-1c in the cytosol and preventing its nuclear translocation, ZnO NPs ameliorated the hepatic steatosis accompanied by insulin resistance, thus hindering lipogenesis [143].

Humans absorb zinc from the small intestine [144], so impaired absorption caused by short bowel syndrome, Crohn’s disease, and severe diarrhea can cause zinc deficiency [145,146]. In exocrine pancreatic insufficiency, zinc deficits can ensue [147]. Patients who have pancreatectomy or pancreaticoduodenectomy may develop post-operation NAFLD, according to studies [148,149,150]. Low-protein diets, high cadmium levels, and phytic acid in fiber-rich foods such as beans, nuts, grains, and seeds [151] can reduce zinc absorption from the gut. The meta-analysis [101] found no significant difference in zinc intake, particularly from plant sources, between NAFLD patients and healthy controls. NAFLD patients’ diets and intestinal changes need further study, which will establish whether blood zinc levels are lower due to poor zinc absorption or increased zinc requirements, which may be linked to insulin secretion, inflammation, or liver dysfunction [101]. Several investigations found that Zn supplementation did not change serum or hepatic Zn levels. This shows a slight zinc homeostasis impairment in the NAFLD model being examined. Existing data show that peripheral tissues rapidly absorb Zn after intestinal absorption, with the pancreas, muscle, prostate, and bone seeing the most accumulation [152,153,154]. 

Lastly, zinc deficiency (below 70 lg/dL) was associated with greater risks of HCC and extra-hepatic malignancies in Japanese NAFLD patients (*p* < 0.001 and 0.026), respectively [102]. Zinc supplementation reduced systolic blood pressure in a meta-analysis of nine randomized clinical studies. It had little effect on diastolic blood pressure [155]. In addition, following the Dietary Approaches to Stop Hypertension (DASH) diet for eight weeks improved weight, BMI, ALT, ALP, triglycerides, insulin metabolism markers, inflammatory markers, GSH, and MDA in NAFLD patients [156]. Zinc supplementation prevents NAFLD-induced liver cobalt loss and raises liver iron levels while reducing hepatic aluminum (Al) and serum vanadium (V). Zn did not affect NAFLD-induced liver I, Mn, and Se depletion [152].

### 3.4. Selenium

Selenium is an essential mineral that plays an important role in redox equilibrium, thyroid hormone metabolism, and defense against OS and inflammation [157]. Selenium is received through diet, with significant quantities found in meat, fish, eggs, dairy products, bread, and cereals [158]. There is a large disparity in global selenium consumption [155]. In China, plasma selenium concentrations range from 22 to 550 μg/L, representing both the lowest and highest intakes observed [159,160,161]. It is critical to acknowledge that selenium exhibits a narrow spectrum between being physiologically necessary and being toxic [162]. 

Several animal experiments and clinical trials have been conducted to evaluate the possible impact of selenium on NAFLD, with variable results. Multiple studies have shown that including selenium supplements in diets can improve the functioning of selenoproteins inside the liver, leading to improvements in liver steatosis, damage, and fibrosis in mouse models of NAFLD [163]. In animal research by Wang [164] and Mousavi et al. [165], higher doses of selenium were observed to lower hepatic steatosis, HOMA-IR, LDL/HDL-c, and TC/HDL-c ratios. On the contrary, epidemiological studies on human subjects have found a link between higher blood selenium levels or higher dietary selenium intake and a higher prevalence of NAFLD [157,166]. Elevated blood selenium levels have the potential to cause hyperglycemia, hyperinsulinemia, and hyperlipidemia, as well as the activation of lipogenesis-related proteins [167,168]. The established dietary guideline for selenium intake among adults in the United States is 55 μg/day per individual [169]. According to 2009–2010 NHANES, adult men in the US have an average daily intake of selenium of triple the recommended amount [170]. It is worth noting that following a vegan or vegetarian diet has been associated with a lower selenium intake [171,172]. Furthermore, the link between selenium levels and glucose regulation extends beyond the mere control of raised blood sugar levels to include the possibility of lowered blood sugar levels in selenium-deficient individuals [173]. In patients with T2DM and NAFLD, there is a negative relationship between selenium consumption and insulin resistance. This beneficial effect, however, stops when the dietary intake of selenium exceeds a threshold of 112 μg/day in a 70 kg individual [174]. SELENOP is a protein that regulates insulin signaling and glucose management. It is an antioxidant with anti-ER effects on the pancreas and blood vessels. Larger doses of SELENOP, on the other hand, lead to the onset and progression of insulin resistance in the liver, adipose tissue, and skeletal muscle [175,176]. In a cross-sectional study of Chinese individuals [166], increased levels of selenium were shown to be positively linked with higher levels of triglycerides and LDL cholesterol. Furthermore, participants with NAFLD had significantly greater selenium contents in their plasma compared to those without NAFLD (270.2 μg/L and 192.5 μg/L, respectively; P 0.01). These findings imply that excessive selenium consumption may alter molecular targets involved in energy metabolism in skeletal muscle and visceral adipose tissue [177]. These significant findings suggest that high selenium levels may have a direct effect on the development of hepatic steatosis. 

Selenium compounds, notably selenite, have been shown to promote the development of potentially hazardous ROS [178,179]. Increased ROS generation can then cause lipid peroxidation by activating stellate cells, resulting in hepatic inflammation [180]. Multiple in vivo animal investigations have supported the idea that dietary selenium may cause OS [181,182,183,184]. This OS not only causes hepatic steatosis, but also contributes to fibrosis progression in NAFLD [185,186]. Nonetheless, the present body of evidence on the ability of selenium to cause OS is equivocal, as other findings imply an opposing impact [187]. The analysis of a cohort of 33,944 people from the Third NHANES [188] showcases that, as serum selenium levels increased, the odds ratio of advanced liver fibrosis (defined as fibrosis score greater than 0.676) decreased significantly. The antioxidant properties of selenium have the potential to explain the observed result. NAFLD is caused by the accumulation and oxidation of fatty acids in the liver, which results in antioxidant depletion and the production of excessive ROS. This OS eventually deteriorates hepatocyte structure and function, aggravating the course of NAFLD [189,190]. Increased GPx activity is one of the mechanisms by which selenium exerts its hepatoprotective effects. Reduced GPx activity has been linked to hepatic inflammation and the development of liver fibrosis [191,192]. Selenium can decrease the activities of metalloproteinases, TNF-α, IL-6, TGF-β1, and a variety of other cytokines and growth factors involved in the development of NAFLD. This inhibition could help to reduce the hepatic inflammation and fibrosis associated with NAFLD [191,193,194,195]. There is a theory that administering selenium supplementation in situations of chronic inflammation helps refill depleted levels of selenium in the liver. This replenishment, in turn, may increase the production of selenoproteins, restoring Se levels in the bloodstream. As a result, C-reactive protein production may be decreased, resulting in a reduction in the inflammatory response [196]. MDA is an example of lipid peroxidation [158].

### 3.5. Magnesium

Magnesium (Mg) is an important cation in biological systems with functions such as activating or inhibiting enzymes, regulating cell cycle and differentiation [197], preserving genomic stability [198], aiding protein synthesis, and maintaining electrolyte balance [199]. Dietary sources of magnesium include whole grains, leafy greens, legumes, and nuts [200]. Approximately 20% of people in developed countries have a magnesium deficiency, possibly due to inadequate consumption [201]. A magnesium deficiency often leads to conditions like diabetes, cardiovascular disorders [202], and kidney diseases [203]. 

A cross-sectional study in Brazil [204] found that people with metabolic syndrome, which included obesity, insulin resistance, and hepatic steatosis, had lower levels of magnesium in their blood and cells. Insulin resistance is widely recognized as the primary mechanism in the pathophysiology of NAFLD [205]. Magnesium is important for insulin action, and insulin regulates intracellular magnesium concentration [206]. A possible link exists between lower Mg levels and decreased tyrosine kinase activity, which can result in the reduced insulin’s ability to facilitate glucose absorption in adipose tissue and skeletal muscle [207]. Magnesium aids glucose transport in the insulin signaling pathway [208]. Low magnesium levels may contribute to insulin resistance by reducing glucose use in cells. A Canadian cohort study found that higher dietary magnesium intake is linked to lower insulin resistance levels measured by HOMA-IR [209]. NAFLD patients may have low serum magnesium levels, which is due to absorption and transportation issues caused by intestinal edema and low albumin levels [210]. Liver illnesses may be caused by low Mg levels in the blood and liver tissue, leading to issues like impaired mitochondrial activity, inflammation, protein kinase C translocation problems, and OS [210]. A Mg shortage can worsen inflammation or OS in NAFLD. Decreased Mg intake can increase chronic inflammation, affecting the severity of pathological disorders. Low magnesium intake causes reduced extracellular magnesium levels, leading to macrophage activation and calcium influx into cells with increased secretion of pro-inflammatory cytokines [200]. In an animal experiment [211] conducted with mice, their Mg consumption was restricted, which led to hepatocyte enlargement and lipid accumulation around hepatocytes in their livers. Enlarged hepatocytes in human NAFLD may suggest early ballooning cell degeneration. Among 33 subjects suffering from NASH, their high dietary total antioxidant capacity is believed to reduce hepatocyte ballooning presence [212]. OS is an important factor in fatal hepatocyte injury [186]. Magnesium deficiency can increase cellular susceptibility to oxidative harm and ROS [213], leading to oxidative damage and chronic inflammation, which are linked to disease progression [214,215]. In vitro studies have shown that adding magnesium increases the production of the antioxidant enzyme SOD [216]. This enzyme neutralizes oxygen free radicals, protecting cells from superoxide free radicals. 

A study by Eshraghian et al. [217] showed a link between low magnesium levels and hepatic steatosis. The study found that people with steatosis levels above 5% had lower serum magnesium levels (1.91 ± 0.51 mg/dL) compared to those without steatosis (2.23 ± 0.31 mg/dL) (*p* = 0.002). This method helped establish a threshold value of 2.05 mg/dL for serum magnesium, indicating the presence of steatohepatitis in liver biopsies (sensitivity = 61%; specificity = 86%; AUC = 0.773; *p* = 0.001). This leads to the conclusion that serum magnesium levels can differentiate hepatic steatosis from steatohepatitis [217]. A US study on young adults, with a subsequent follow-up period of 25 years [218], found that higher magnesium intake was linked to a lower risk of NAFLD. In comparison to those in the lowest quartile of total magnesium consumption, those in the highest quintile exhibited a 55% reduction in the likelihood of NAFLD [OR = 0.45, 95% CI (0.23, 0.85), *p* = 0.03]. Whole grains, a major source of Mg, are consistently linked to a lower risk of NAFLD (*p* = 0.02) [219], while low magnesium diets, like gluten-free diets, may increase the risk of the disease. A study that used data from NHANE III to examine the link between Mg intake and NAFLD risk revealed an inverse correlation between the variables [220]. Lu et al. [218] found that higher magnesium intake in adulthood is linked to a lower risk of NAFLD in middle-aged Americans. Additionally, individuals with NAFLD are more susceptible to magnesium deficiency [221]. There is a correlation between magnesium intake and inflammatory biomarkers like TNF-α [222], VCAM-1 [223], and CRP [214]. Magnesium intake is important for people with NAFLD. A study found that patients with NAFLD who developed HCC had lower average serum magnesium levels (0.769 ± 0.131 mmol/L) compared to those who did not develop HCC (0.789 ± 0.125 mmol/L; *p* = 0.003). In addition, NAFLD patients with high serum magnesium (>0.864 mmol/L) had a significant 27% reduction (95% CI, −44 to −4) in HCC incidence [224]. 

An effective magnesium treatment is magnesium isoglycyrrhizinate (MGIG), derived from 18α-glycyrrhizic acid. MGIG exhibits anti-inflammatory properties and serves as a hepatoprotective drug by safeguarding hepatic cells against tissue damage, enhancing liver function, and suppressing inflammatory reactions inside the liver [225,226,227,228,229]. MGIG treatment preserves cell viability, reducing cell apoptosis and lipid accumulation caused by oleic acid (OA) [230]. MGIG can reduce lipid accumulation by suppressing the expression of metabolic enzymes (GPATs and DAGTs) that are involved in the manufacture of cellular triglycerides [231]. More research showed that MGIG treatment of hepatic cells that were overloaded with lipids greatly decreased the levels of inflammatory biomarkers such as NF-κB, IL-6, TGF-β, and bFGF in both mRNA and protein. MGIG effectively inhibits UPR, reducing inflammatory cytokine production and protecting hepatic cells against NAFLD-induced injury [232]. Administering MGIG therapy at 30 mg/kg, along with atorvastatin, effectively reduces obesity and hepatic edema caused by a high-fat diet (HFD) [233]. Mice treated with MGIG had fewer hepatic vacuoles and a lower degree of inflammatory infiltration than the HFD group [234]. Lastly, the same study observed a significant decrease in TLR4 expression after MGIG therapy, which suggests a link between MGIG’s anti-inflammatory properties and its ability to inhibit lipidation. The study urges that targeting hepatic TLR4 could be important for modulating energy balance in hepatic steatosis. Another potential treatment for NAFLD is CIRRHOS, which contains 2 g of L-carnitine and 150 mg of magnesium. During the trial, the therapy group showed a 25% reduction (*p* = 0.9) in AST levels and a 20% reduction (*p* = 0.1) in ALT levels compared to the baseline levels. However, therapy did not cause significant changes in CRP levels, insulin levels, lipid profiles, or LS [234]. 

The correlation between magnesium and calcium is important and needs investigation. High calcium intake leads to increased magnesium excretion [235,236]. Increased renal excretion or decreased gastrointestinal absorption of magnesium can also cause hypomagnesemia [237]. A positive correlation was established between the calcium-to-magnesium ratio in the diet and the risk of developing NAFLD, with the study accounting for potentially confounding variables such as age, gender, BMI, alcohol, smoking, diabetes, physical activity, calories, fiber, carbs, fat, and protein [238]. There is a stronger correlation between Mg consumption and a decreased risk of pre-diabetes and fatty liver disease when Ca intake is below 1200 mg/day. Excessive calcium intake may hinder the beneficial effects of magnesium [220]. 

### 3.6. Vitamin A

Regarding NAFLD, the function of Vitamin A (VA), also known as retinoic acid, has not been thoroughly investigated. Studies have shown, however, that patients with NAFLD have a deficiency in vitamin A relative to non-diabetic and diabetic non-NAFLD patients [239,240,241,242,243,244]. A deficiency of VA, a lipid-soluble vitamin known for its antioxidant properties [245], could potentially exacerbate liver injury since NAFLD pathology is associated with OS [246]. Carotenoids, as well as retinyl esters derived from retinitis-rich animal products (e.g., eggs, fish, and liver), are potential sources [247]. A sufficient daily intake of 700–900 µg for humans and hepatic storage of 80% in a healthy individual is necessary to sustain plasma retinol levels at approximately 2 µmol/L [248,249]. HSCs are tasked with the storage of VA within the liver. Upon activation, HSCs deplete their intracellular vitamin A reserves, resulting in their conversion into myofibroblasts that produce collagen [250,251]. Several physiological processes, including vision, cell proliferation and differentiation, immune regulation, embryogenesis, glucose and lipid metabolism, and embryogenesis, rely on VA. In a study examining transgenic mice with defective retinoic acid receptors in the liver, Yanagitani et al. [252] observed that HCC and NASH developed after four and twelve months, respectively. In the morbidly obese population [253], an inverse correlation was observed between NAFLD and the concentrations of AST (aspartate aminotransferase), ALT, and serum retinol. In contrast to the above-mentioned investigations, Bahcecioglu et al. [254] discovered that patients afflicted with simple steatosis and NASH exhibited elevated levels of serum retinol in comparison to their healthy counterparts. The majority of prior research examining the association between retinol and NAFLD concentrated on animal subjects [252,255,256] or obese populations [244,253] with small sample sizes, thereby limiting the applicability of these findings to the general population. In this middle-aged and elderly Chinese population, Xiao et al. [257] demonstrated that patients with more severe degrees of NAFLD had higher concentrations of serum vitamin A after a three-year follow-up. Potential contributors to the observed outcomes include elevated RBP4, TG, insulin resistance, and BMI. Elevated concentrations of circulating VA may not be advantageous for the prevention or treatment of NAFLD, according to these results. 

Patients diagnosed with NAFLD and NASH exhibit reduced concentrations of serum retinoic acid (RA). In patients with NAFLD and NASH, decreased RA concentration is associated with intrahepatic triglyceride concentration, the severity of hepatic steatosis, and liver injury [258]. In addition, indicators of liver injury and adiposity (intrahepatic triglyceride and transaminase levels) are negatively correlated with serum RA levels. This negative correlation provides further support for the notion that vitamin A may influence lipid and glucose metabolism in the liver [240,241]. The observed phenomenon can be attributed to retinoic acid’s capacity to upregulate the expression of genes involved in fatty oxidation promotion within the liver. These genes include uncoupling protein 2, proliferator-activated receptor alpha (PPARα), fibroblast growth factor 21 (FGF21), and carnitine palmitoyltransferase I (CPT1) and uncoupling protein 2 (UCP2) [247]. 

A research investigation was carried out on wild-type mice that were provided with a high-fat diet (HFD) supplemented with RA. The study documented several beneficial effects, including a reduction in hepatic intracellular triglyceride, serum ALT, and aspartate AST concentrations, subcutaneous and visceral fat content, and apparent hepatosteatosis reversal. Research conducted in vivo demonstrated that RA reduced adiposity not only in the liver but also in adipose tissue by increasing triglyceride hydrolysis and fat oxidation [259,260,261]. Recent discoveries that demonstrate a novel transcriptional regulatory cascade governing hepatic lipid metabolism in a murine model have identified RA signaling as a potential new therapeutic strategy for NAFLD [262]. Determined lately as a member of the lipocalin family, retinol-binding protein 4 (RBP4) serves as the blood’s specific carrier protein for vitamin A [263]. The liver has the highest RBP4 expression, followed by adipose tissue [264]. Research conducted on animals indicates that insulin resistance can be induced by transgenic over-expression of human RBP4 or injection of recombinant RBP4 into wild-type mice; conversely, deletion of the RBP4 gene results in increased insulin sensitivity [265]. Elevated levels of circulating RBP4 have been linked to obesity, insulin resistance, impaired glucose tolerance, and T2DM in humans, according to a few studies [266,267,268,269]. Certain studies have shown that NAFLD patients have considerably elevated RBP4 levels in comparison to healthy control individuals [269,270]. However, other studies have not identified any correlation between NAFLD and RBP4 levels [271,272]. There were no statistically significant differences in circulating RBP4 levels between NAFLD patients and controls, according to a meta-analysis [273] (SMD]: 0.08; 95% CI: −0.21, 0.38). Hyperglycemia, hepatic enzymatic activity, and hyperlipidemia were diminished in HFD animals due to RBP4 expression downregulation in adipose tissue and the liver [274]. 

Vitamin A and its downstream metabolites have potential therapeutic applications, because these compounds influence hepatic glucose and lipid metabolism through interactions with nuclear receptors including PPARα, PPARβ/γ, PPARγ, and FXR [243]. Furthermore, this class of compounds functions as antioxidants in addition to performing essential roles in lipid metabolic homeostasis for vitamin A and its derivatives. An investigation illustrated that oral supplements containing all-trans-retinoic acid (ATRA) significantly decreased OS and hepatic iron accumulation induced by excess iron. Additionally, this treatment induced an increase in the expression of the hepatic leptin receptor, thereby stimulating leptin signaling in the liver. Furthermore, it restored insulin sensitivity in insulin-resistant mice as well as mice fed an HFHFr diet; these results strongly indicate that ATRA may serve as a viable treatment option for NAFLD-associated insulin resistance [275]. OS contributes to the development of NAFLD and may necessitate increased vitamin A intake for antioxidant functions. A decrease in vitamin A concentration is hypothesized to accelerate the progression of liver disease and increase the risk of developing HCC [244]. 

A study involving 68 class III obese NAFLD [244] patients found that insufficient levels of liver retinol were substantially correlated with the severity of NAFLD. Serum retinol levels were significantly decreased in patients with advanced liver fibrosis, according to Coelho et al. [246] (*p* = 0.002). Significantly, a lack of dietary vitamin A was observed in nearly all the participants in the sample. Insufficient consumption of VA results in the depletion of retinol reserves and potentially aids in the advancement of hepatic pathology [246]. Three cross-sectional investigations from the United States [276], China [257], and Korea [277] found a positive correlation between serum VA levels and the severity of NAFLD; this correlation was also influenced by BMI. Several genomic loci have been identified as risk factors for NAFLD, with a genetic variant of PNPLA3-I148M being the most prominent. This heritable factor is strongly associated with NAFLD. It is recognized as a risk factor for disease progression, and HCC is associated with NAFLD. The PNPLA3-I148M genetic variant exhibits reduced hydrolase activity and facilitates the accumulation of triglycerides in the liver. It has been reported that the excessive expression of PNPLA3-I148M induces hepatic steatosis in mice and modifies levels of circulating retinol in humans [278]. Based on a cross-sectional analysis utilizing data from the 2017–2018 NHANES cycle involving 3537 participants [279], adult patients with NAFLD status may have a positive correlation with serum retinol levels, while liver fibrosis may have a negative association with serum retinol levels. Vitamin A homeostasis dysregulation in the liver could potentially contribute to the development of NAFLD [280]. Deficiencies in vitamin A homeostasis that occur during the onset and progression of NAFLD may have an impact on lipid accumulation in the liver [281]. This causes retinol to be released excessively and to accumulate in the serum [282]. An extreme reduction in vitamin A levels during the progression of NAFLD to advanced fibrosis results in a severe deficiency of vitamin A in the liver, subsequently causing a decline in serum retinol concentrations [279]. With the ability to inhibit hepatic cell transformation, suppress the proliferation of hepatoma cells, prevent the production of pro-inflammatory cytokines in macrophages, mitigate inflammatory reactions, and provide antioxidant protection against OS, which has been implicated in the development and progression of NAFLD, the VA and its derivative retinoids play crucial roles in regulating cellular growth and differentiation [283,284,285,286]. 

### 3.7. Vitamin B

The Vitamin B group consists of eight vitamins: B1—thiamin, B2—riboflavin, B5—pantothenic acid, B6—pyridoxine, B7—biotin, B9—folic acid and B12—cyanocobalamin. Vitamin B3 is a precursor to coenzymes involved in lipid metabolism—nicotinamide adenine dinucleotide (NAD) and nicotinamide adenine dinucleotide phosphate (NADPH) [287]. In a study on conventional uses and new insights on vitamins, its supplementation has been shown to increase redox potential, lower hepatic cholesterol content, and inhibit the gain in liver weight in an obesogenic diet-induced rat model of NAFLD. It has also displayed a protective effect on pre-existing hepatic steatosis, which supports the possibility of vitamin B3 being used to treat NAFLD. Niacin also lowered plasma triglyceride concentration and hepatic fat content, and improved liver enzymes in dyslipidemic patients [288]. Vitamin B12 is found in humans in two forms: methyl cobalamin and 5’-deoxyadenosylcobalamine. They are stored in the liver. Supplementation with Vitamin B12 among patients with NAFLD has been shown to improve the serum levels of homocysteine (HCY), and fasting blood glucose and serum levels of MDA were significantly improved in the trial group who received Vitamin B12 [289]. Vitamin B12 absorption is often affected in patients with NAFLD. Low serum B12 levels are correlated with high HCY levels. A study on Vitamin B12 and HCY levels in NAFLD patients has shown that HCY levels were significantly higher in patients with NAFLD. This research suggests that HCY levels could be a potential marker for liver damage [290]. This is supported by another study, which concluded that serum Vitamin B12 levels were much lower in patients with NAFLD. Consequently, low Vitamin B12 levels may be associated with NAFLD, especially in grade 2 to grade 3 hepatosteatosis [291]. A study researching the association between protein and B12 intake and liver steatosis found a positive correlation between the above-mentioned factors and suggested a pathogenic role of Vitamin B6 in the increase in hepatic steatosis [292]. Findings of a study on the relation between the index of nutritional quality and NAFLD suggest that subjects who adhere to a healthier and nutrient-rich diet, including Vitamins B1, B2, B12, and B3, are at a lower risk of NAFLD compared to those who follow an unhealthy and nutrient-poor diet [293].

### 3.8. Vitamin C

OS causes inflammation in liver cells and, in turn, leads to dyslipidemia. Vitamin C (ascorbic acid) is a powerful antioxidant [282], which suggests that it may contribute to NAFLD prevention. Ascorbic acid is also connected to the regulation of adiponectin, which decelerates lipid accumulation in the liver, and inflammation of hepatic cells, possibly lowering the risk of NAFLD [294]. A cross-sectional study on the association between dietary Vitamin C intake and NAFLD demonstrated a moderate inverse association between dietary Vitamin C intake and NAFLD in middle-aged and older adults, especially for the male population and non-obese population [295]. Another study, conducted among the adult population of the United States, supports this [296]. Vitamin C supplementation has also been shown to cause a significant improvement in the liver fibrosis score of NASH patients [246]. Vitamin C deficiency was found, by a study performed on guinea pigs, to exacerbate dyslipidemia and liver damage, while its increased intake led to a decrease in dyslipidemia severity and accumulation of lipids in hepatocytes [297]. The combination of Vitamins C and E together with atorvastatin (20 mg) is suggested to effectively reduce hepatic steatosis by 71% in patients with NAFLD [298]. The effects of the combination of Vitamins C and E on individuals afflicted by NAFLD have also been evaluated in other studies due to their antioxidant properties, and the research has supported its effectiveness [295].

### 3.9. Vitamin D

A total of 55% of NAFLD sufferers are Vitamin D-deficient [299]. There is a confirmed association between low Vitamin D levels and obesity and IR [300]. IR causes OS and lipotoxicity and serves as a precursor to NAFLD [301]. Vitamin D deficiency is also confirmed to contribute to a higher risk of hepatic steatosis in morbidly obese patients [302]. The relationship between Vitamin D and NAFLD has been studied in various demographics and an inverse association between the concentration of Vitamin D and NAFLD was confirmed [303,304]. Vitamin D deficiency causes the activation of Toll-like receptors which leads to severe liver inflammation and causes OS. Vitamin D supplements were found to be able to reverse the inflammation caused by NAFLD-related hepatic injury, which suggests the possible use of Vitamin D in treating NAFLD [239,305]. Another study has found that Vitamin D supplements may improve NAFLD by reducing inflammation and improving the lipid profile, but the exact mechanism of inflammation reduction in NAFLD is still unknown. Vitamin D in active form may directly affect NAFLD development [306]. However, excessive Vitamin D supplementation may lead to hypercalcemia, which is a risk factor for NAFLD [307,308]. 

### 3.10. Vitamin E

Vitamin E occurs in eight natural forms: four tocopherols (α, β, γ, δ) and four tocotrienols (α, β, γ, δ). The most abundant one is α-tocopherol, and it is also the one that is the most effective in inhibiting lipid oxidation [247]. Of all known vitamins, evidence points to Vitamin E having the most therapeutic potential in NAFLD [309]. As an antioxidant, it reduces OS and decelerates the pathogenesis of NASH. It lowers the inflammatory response by increasing the expression of adiponectin and suppresses the expression of several cytokines, functions as a scavenger of certain radicals, and possesses anti-apoptotic properties [247]. Studies have also shown that Vitamin E prevents the accumulation of lipids in the liver by downregulating a membrane transporter protein responsible for transporting fatty acids into the liver [310]. Due to these properties, the American Association for the Study of Liver Disease (AASLD) and the European Association for the Study of the Liver (EASL) recommend the supplementation of Vitamin E (800 IU per day) in non-diabetic adults afflicted with NASH [309,311]. Vitamin E supplements are also commonly prescribed to patients with NAFLD [312]. A study conducted on a rat model for NASH resulted in Vitamin E mitigating lipid peroxidation [313]. Trials performed on both adults and children showed a significant improvement in steatosis and inflammation in patients who were treated with vitamin E for 96 weeks compared to the placebo group [314,315]. This was supported by another study which found that children with NAFLD have an insufficient amount of Vitamin E in their diet, suggesting that it may contribute to the pathophysiology of NAFLD [316].

### 3.11. Carotenoids

Carotenoids are a class of fat-soluble pigments that possess antioxidant properties [317]. They are mainly stored in the liver [318]. Among them, astaxanthin, lutein, β-carotene, and fucoxanthin were researched in relation to NAFLD. Astaxanthin is a carotenoid with a long chain with conjugated double bonds and keto moieties on each ionone ring. It is a potent antioxidant that scavenges mostly peroxyl radicals, protecting fatty acids and biological membranes against lipid peroxidation, which suggests that it may alleviate NAFLD-related OS [319,320]. Studies conducted on a mouse model showed that astaxanthin decreases lipid accumulation in hepatocytes as well as lowers inflammation and fibrosis in hepatic tissue when combined with Vitamin E [321]. 

Lutein and its isomer zeaxanthin belong to the xanthophyll group of carotenoids and have two hydroxyl groups in their structure [322]. They were shown to enhance immune function and lower the risk of cancer, cardiovascular disease, and OS-related diseases, such as NAFLD [323,324,325]. A study on NAFLD in Chinese adults has demonstrated an inverse association between the concentration of lutein and zeaxanthin and NAFLD prevalence [326]. The relation between lutein and cholesterol-induced liver damage was tested on a guinea pig model, which resulted in lutein lowering free hepatic cholesterol by 43% and mitigating lipid peroxidation. It has also been suggested that lutein may have anti-inflammatory properties [327]. This was supported by a similar study conducted on the same model [326]. Zeaxanthin was also tested independently on Mongolian gerbils and was shown to reduce fibrosis and lower lipid hydroperoxides [328]. 

β-carotene is a retinol precursor and antioxidant that is found in certain fruits and vegetables and has been found to have a strong anti-inflammatory effect [329,330]. Higher sodium intake increases the odds of NAFLD by upregulating inflammation. Dietary β-carotene was suggested to attenuate this association by downregulating inflammation [331]. It was also found to alleviate hepatic steatosis and fibrosis [332]. β-carotene has been proven to support the treatment and prevention of NAFLD and can enhance the therapeutic effect of other drugs on NAFLD, such as rosuvastatin, which was found to be more effective in combination with β-carotene than alone [333]. 

Its acyclic isomer, lycopene, is one of the most potent dietary antioxidants. This is due to its long, acyclic polyene chain, which gives it a higher singlet oxygen quenching capacity than that of β-carotene and α-tocopherol [334,335]. It also possesses other qualities that may prove useful in preventing chronic diseases, such as regulation of gene expression, gap-junction communication, anti-proliferative capacity, and hormone and immune modulation [334,336,337]. Those properties make it one of the most studied carotenoids regarding NAFLD. Lycopene is most commonly found in tomatoes—tomatoes themselves and tomato-based products are the most common sources of lycopene in the human diet and account for more than 85% of the dietary intake of this carotenoid in North America. Epidemiological studies have demonstrated an inverse relationship between tomato intake and several chronic diseases, which suggests that lycopene may be at least one of the factors that lead to the prevention of chronic diseases by tomato consumption [334,336,338]. Other studies on animal models support the notion that lycopene reduces high-fat diet-induced OS and liver damage, suggesting a protective role of this carotenoid in the development of NAFLD [339,340]. There have also been attempts to research the use of lycopene in treating NAFLD instead of prevention. The results suggest that bioactive antioxidant components such as lycopene could be used in addition to dietary interventions to accelerate the recovery from and reversion of high-fat diet-induced liver damage [341]. 

Fucoxanthin is a carotenoid present in the chloroplasts of brown seaweeds. It possesses strong anti-inflammatory activity and cancer prevention through its antioxidant activities [342]. High-stability fucoxanthin (HSFx) also exhibits anti-obesity properties [343,344]. Due to its antioxidant, anti-inflammatory, and anti-lipemic properties, HSFx has great potential for NAFLD treatment. HSFx reduces hepatic steatosis and fibrosis in patients with NAFLD and is suggested to attenuate NAFLD-induced inflammation and adipogenesis [345].

## 4. Macronutrients Involved in NAFLD

### 4.1. Carbohydrates

A dietary study in NAFLD patients showed that a diet solely restricted in carbohydrates had a greater reduction in liver fat when compared with a calorie-restricted diet regardless of overall weight loss [346]. Research conducted on the interaction between NAFLD severity, high carbohydrate diet, and gut microbiome implied that the consumption of a high carbohydrate (HC) diet is associated with alteration in the gut microbiome (Enterobacteriaceae, Ruminococcaceae, and Veillonellaceae), defective glucose homeostasis, and the upregulation of hepatic de novo lipogenesis. Sources of lipid biosynthesis dysregulation include de novo lipogenesis [DNL] from carbohydrates, adipose tissue lipolysis leading to the derivation of free fatty acids (FFAs), as well as excessive dietary fat intake in the form of intestinal chylomicrons [347]. DNL contributes up to 26% of the total triglyceride synthesis in NAFLD patients with hyperinsulinemia, while serum-derived non-esterified fatty acids account for about 59%, and the diet accounts for 15% [348]. These changes altogether lead to the progression of NAFLD [349]. Moreover, clinical evidence suggests that ketonic, simple sugar fructose, through increased lipogenesis and impaired fat oxidation, precipitates fat accumulation in the liver [350]. Carbohydrate restriction is said to have an independent effect on resolving hepatic lipid accumulation. After one year of carbohydrate-restricted intervention, the proportion of individuals with steatosis was reduced by 20%, with improvements in the percentage of individuals without any signs of fibrosis (from 18% at baseline to 33%) [351]. Additionally, a meta-analysis of 10 studies reported that low carbohydrate intervention significantly reduces hepatic lipid content [352]. In another study, Mediterranean low-carbohydrate diet introduction resulted in a significantly greater reduction in liver, pancreas, and pericardium fat stores, as well as triglycerides [353]. The additional study introduced 10 obese patients with high liver fat to a strictly ketogenic eucaloric diet for 14 days. The carbohydrate intake was reduced to 4%, the fat intake increased to 72%, and the protein intake increased to 24%. In two weeks, the liver fat content had decreased in all participants by approximately 44% with minimal weight loss. The decrease in liver fat was accompanied by a reduction in de novo lipogenesis and an increase in fatty acid oxidation [354]. In conclusion, the reduction in carbohydrate intake and the modification of carbohydrate quality play important roles in nutrition therapy in NAFLD patients. 

### 4.2. Fat

Among fatty acids, we distinguish saturated and unsaturated ones. Saturated fatty acids are involved in hepatic steatosis, and thus in NAFLD progress, whereas moderate amounts of unsaturated fatty acids are believed to help with preventing said condition development. 

Extra virgin olive oil (EVOO), an example of unsaturated fatty acid, helps to cease the advancement of NAFLD and significantly reduces the liver fat content. Animal studies reported that the beneficial effect of the Mediterranean diet is believed to be due to its rich EVOO content. It is said to significantly reduce liver fat and resolve the pro-inflammatory state. EVOO is concentrated with polyphenols, such as hydroxytyrosol, that salvage insulin receptor signaling by modifying ER stress in adipose and liver tissue [355]. Furthermore, EVOO improves ultrastructural damage and lipid deposit in the liver as well as suppresses the expression of the lipogenesis genes. The n-3 long-chain polyunsaturated fatty acids eicosapentaenoic acid (EPA) and docosahexaenoic acid (DHA) may use antisteatotic and antioxidant responses in the prevention or resolution of inflammation [356]. Previous studies suggested that supplementing long-chain omega-3 fatty acids independently leads to liver fat content reduction [357]. Moreover, this beneficial effect has been reported to be unrelated to weight loss or other dietary changes. When it comes to long-chain omega-3 fatty acids, there are possibly four biological mechanisms involved: the influence of the PPAR (peroxisome proliferator-activated receptors) system, and with this the promotion of the fatty acid oxidation in the liver; the suppression of the de novo lipogenesis of carbohydrates in hyperinsulinemic states (through inhibiting the expression of lipogenic transcription factors SREBP-1C and ChREBP); the bile acids release promotion, that contributes to liver fat and cholesterol secretion; the mediation of adiponectin production, which strengthens adipocytes integrity and reduces their susceptibility to inflammation [358]. A different study suggested that high dietary cholesterol intake leads to a progression of steatosis, steatohepatitis, fibrosis, and hepatocellular cancer in mice. Said induction was connected to gut microbiota dysbiosis. Mucispirillum, Desulfovibrio, Anaerotruncus, and Desulfovibrionaceae occurrence increased, while Bifidobacterium and Bacteroides depleted in high-fat/high-cholesterol diet-fed mice. The results were corroborated in human hypercholesteremia patients. Atorvastatin (cholesterol-lowering drug), restored cholesterol-induced gut microbiota dysbiosis and prevented NAFLD development [359]. Dietary cholesterol-induced gut bacterial metabolites changes, with the increase in taurocholic acid and decrease in 3-indolepropionic acid. Germ-free mice exposed to contact with stools from mice fed HFHC manifested hepatic lipid accumulation, inflammation, and cell proliferation [360].

In recent research, scientists examined the connection between metabolic response in overweight patients and the source of their excessive calorie intake. Said excess was provided by saturated, unsaturated fats or simple sugars. The above-mentioned dietary choices lead to the increase in increased intrahepatic triglyceride content (NAFLD), with an unsaturated fats diet inducing a lesser increase in intrahepatic triglyceride content, when compared to the saturated fats diet. The saturated fats diet increased lipolysis as well as multiple plasma ceramides, which are an independent risk factor of cardiovascular disease, not connected to LDL-cholesterol levels [361]. 

Mitochondrial dysfunction is believed to play a great role in the development of NAFLD and saturated fatty acids change both mitochondrial membrane structure and their function. An overabundance of saturated fatty acids leads to not only liver steatosis but also the depletion of the respiratory transport chain functioning in the mitochondria. The decrease generates excessive production of ROS and cellular damage, with reactive inflammation, apoptosis, and eventual liver fibrosis [362]. 

### 4.3. Proteins

The mobilization of liver fat is independently increased by protein intake. This effect may be altered by branched-chain amino acid (BCAA) and methionine content in the diet [363]. The cause of BCAA occurrence in NAFLD may be obesity, insulin resistance, increased protein catabolism in muscles, or impairment in tissue metabolism. Scientists compared two groups of patients, namely without obesity (NAFLD-NO) and with obesity (NAFLD-Ob), and whether they could observe any changes in plasma AAs compared to controls. Levels of the AAs involved in glutathione synthesis, such as glutamic acid, serine, and glycine, are often found to be altered in NAFLD and may be correlated to its severity. A total of 44 subjects with NAFLD, and no diabetes, were measured their AA values. A liver biopsy, spectrometry, and insulin resistance assessment glutamate–serine–glycine index were performed and compared to liver histology. NAFLD- Ob subjects showed a broader variety of increased AAs in their profile, whereas alanine, glutamate, isoleucine, and valine, but not leucine, were increased in NAFLD-NO subjects compared to controls. Hepatic insulin resistance was associated with the glutamate–serine–glycine index, as well as glutamate and tyrosine. Moreover, said index related to liver enzymes, especially gamma-glutamyltransferase, was able to distinguish advanced fibrosis (stages F3 and 4) from its/initial/earlier forms (stages F0, F1, and F2), with no correlation to BMI. Increased BCAAs plasma levels were associated with inflammation observed in liver biopsy [364]. 

**Figure 2:**
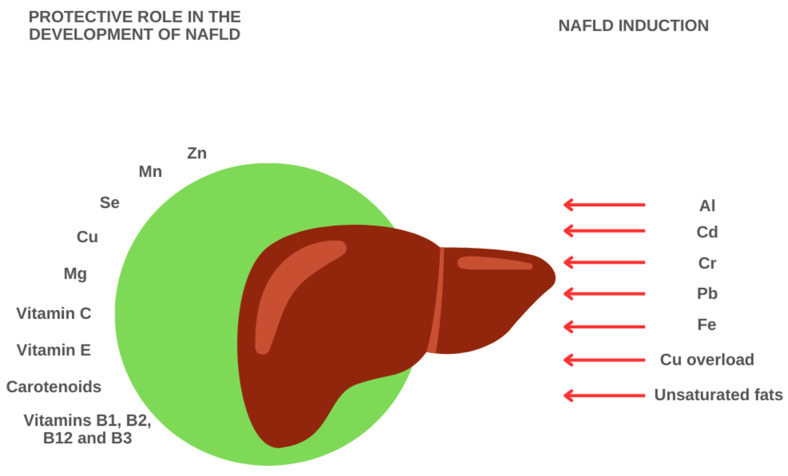
Micro- and macronutrients involved in NAFLD induction and protection against its development (the figure was created in Canva).

Supplementation with 60 g of whey protein per day, during an otherwise ad libitum diet, causes liver fat content to decrease by 21%. Evidence to this hypothesis may be found through a six-week randomized controlled study conducted by the Institute in Potsdam, where an eucaloric, protein-rich diet (30% protein, 30% fat, and 40% carbohydrates) was used. German scientists achieved a significant reduction in liver fat content in patients with T2DM and NAFLD when compared with their usual diet. The participants were randomized into two different protein groups, where one received mainly plant as protein sources (e.g., legumes), and the other group mostly animal protein (e.g., meat and milk products). Both groups reported a significant reduction in liver fat. In addition, a more significant decrease was observed in the animal protein group (−48% versus −36%). This substantial resolution of liver fat, in both groups, was associated with the reduction in free fatty acid levels and improvements in lipogenesis markers [365,366]. Peroxisome proliferator-activated receptors (PPARs) reportedly regulate several important biological processes, such as inflammation, lipid, and glucose metabolism. They belong to the nuclear hormone receptor group and are essential regulators of adipocyte differentiation through the expression of markers involved in lipid metabolism, such as phosphoenolpyruvate carboxykinase. As a result, agonists of PPAR-γ as well as dual agonists of two PPARs are believed to be effective for the treatment of NAFLD [367]. Additionally, we recognize G protein-coupled receptors (GPCRs), the cell surface receptors that mediate the function of extracellular ligands. Studies have shown that more than 50 different GPCRs are supposedly expressed in the mouse liver. However, their regulation role in liver metabolism is limited. More research conducted on said receptors could potentially lead to the development of novel drugs for type 2 diabetes, nonalcoholic fatty liver disease (NAFLD), and nonalcoholic steatohepatitis (NASH) [368]. With a high-protein diet being currently one of the most effective treatments for patients with NAFLD, scientists performed research on Drosophila oenocytes, the specialized hepatocyte-like cells. The results show the important role of Ubr1 family protein activation in novel NAFLD treatment strategies. Essential AAs improve hepatic steatosis with the induction of polyubiquitination of Plin2, which is a protein that stabilizes lipid droplets. BCAAs like leucine and isoleucine bind and activate the E3 ubiquitin ligase Ubr1, degrading Plin2, and Ubr1 activity reportedly prevents steatosis in mouse livers and cultured human hepatocytes (Figure 2) [369].

## 5. Conclusions

Nutritional impairments, dietary habits, and supplementation of macro- and micronutrients play an important role in the development of NAFLD. Research showed that the dysregulation of element levels such as iron or copper may contribute to the progression of NAFLD by increased ROS production and OS enhancement. Proper zinc and magnesium intake may be a crucial factor in NAFLD prevention (Table 1). Better glucose metabolism, decreased OS and lipid deposition in hepatocytes, and reduced liver damage are some of the beneficial outcomes of zinc and magnesium supplementation. What is more, both elements contribute to a decreased risk of HCC. The influence of selenium on NAFLD progression is complex. Selenium supplementation may bring benefits in NAFLD therapy; however, further studies are needed to establish appropriate intake levels. Supplementation of Vitamin B group, Vitamin C, Vitamin D, and Vitamin E may also decrease the risk of NAFLD onset. Beneficial outcomes of these compounds’ proper supplementation such as inhibition of liver weight gain, OS reduction, or downregulation of inflammatory processes may suppress liver steatosis and fibrosis. The exact influence of Vitamin A on NAFLD onset is complex. Research showed potential promising effects of retinoids’ application in therapy of NAFLD but further research is crucial to understand their clinical benefits. Macronutrients such as carbohydrates, fat, and proteins also play an important role in NAFLD progression. The reduction in carbohydrate consumption, intake of adequate amounts of EVOO and long-chain omega-3 fatty acids, and proper protein consumption can reduce inflammation in the liver, lipogenesis processes, and liver damage. Despite the in-depth analysis that the authors aimed for in this review, there are still several limitations in our study such as language limitations which impeded the search of articles beyond the English language, limited access to some articles, as well as the empirical aspects such as representativeness of the groups studied in the reviewed papers. Furthermore, what should be also considered is that the results of the animal studies usually do not fully represent the possible findings in the case of studies involving humans and the results should be interpreted with caution. Overall, the imbalance in macro- and micronutrients as well as the conscious supplementation of elements and compounds mentioned above are crucial for proper liver function and prevention of NAFLD onset and progression. However, further studies—both animal and human—are necessary to discover the exact functions of some nutrients and the healthy ranges of the dosages.

## Figures and Tables

**Table 1:** Involvement of chosen trace elements in hepatic inflammation.

Trace Elements in NAFLD		
Element	Role	References
Iron (Fe)	Iron catalyzes the generation of ROS, leading to hepatic inflammation and ultimately tissue deterioration.	[49]
Zinc (Zn)	Zinc supplementation results in reduced hepatic steatosis and damage, reduced lipid deposition in hepatocytes, improvement in glucose metabolism, and insulin signaling.Zinc deficiency leads to elevated ROS levels, increased risk of DNA damage, protein modification, and carcinogenesis.	[106,152]
Selenium (Se)	Selenium supplementation can improve the functioning of liver selenoproteins, alleviating liver steatosis, damage, and fibrosis in mouse models of NAFLD. However, increased levels of selenium were positively linked with higher levels of tri-glycerides and LDL cholesterol. Patients with NAFLD had significantly greater selenium contents when compared to healthy controls.	[163,166,370]
Copper (Cu)	Excessive levels of copper stimulate lipogenesis and lipolysis by the activation of the Nrf2-PPARγ pathway and cellular apoptosis. Copper deficiency is correlated with liver steatosis and impedes the export of the liver’s iron, leading to its accumulation.	[86,371,372]
Magnesium (Mg)	Low magnesium levels may contribute to insulin resistance by reducing glucose use in cells, as well as impaired mitochondrial activity, inflammation, protein kinase C translocation problems, and oxidative stress.	[200,210]
Vitamin A	Vitamin A and its retinoids can inhibit hepatic cell transformation, suppress the proliferation of hepatoma cells, prevent the production of pro-inflammatory cytokines in macrophages, mitigate inflammatory reactions, and provide antioxidant protection against oxidative stress.	[283,284]
Vitamin B	Vitamin B3 is a precursor to coenzymes involved in lipid metabolism—NAD and NADPH. Niacin increases redox potential, lowers hepatic cholesterol content, and has a protective effect on pre-existing hepatic steatosis. It is suggested that Vitamin B6 has a pathogenic role in the increase in hepatic steatosis.	[287,292]
Vitamin C	Ascorbic acid is related to the regulation of adiponectin, which decelerates lipid accumulation in the liver, and inflammation of hepatic cells, which possibly lowers the risk of NAFLD.	[294]
Vitamin D	Vitamin D deficiency causes the activation of Toll-like receptors which leads to severe liver inflammation and oxidative stress.	[303,305]
Vitamin E	Vitamin E is an antioxidant, it reduces OS and decelerates the pathogenesis of NASH. It lowers the inflammatory response by increasing the expression of adiponectin. It prevents lipid in the liver, by downregulating a membrane trans-porter protein.	[247,310]
Carotenoids	Possess strong anti-inflammatory activities and cancer prevention through their antioxidant activities and lycopene is said to reduce high-fat diet-induced oxidative stress and liver damage.	[334,339,342]

## Abbreviations

AASLD	American Association for the Study of Liver Disease
ALT	alanine aminotransferase
BCAA	branched-chain amino acid
BMI	body mass index
COX	cytochrome c oxidase
DHA	docosahexaenoic acid
DIOS	dysmetabolic iron overload syndrome
DMT1	divalent metal transporter 1
DNL	de novo lipogenesis
DOW	deep ocean water
CPT1	carnitine palmitoyltransferase I
EASL	European Association for the Study of the Liver
EGCG	epigallocatechin-3-gallate
EPA	eicosapentaenoic acid
ER	endoplasmic reticulum
EVOO	extra virgin olive oil
FFA	free fatty acid
FGF21	fibroblast growth factor 21
GLUT4	glucose transporter 4
GPCRs	G protein-coupled receptors
GPx	glutathione peroxidase
HC	hepatocellular cells
HCC	hepatocellular carcinoma
HCY	homocysteine
HDL	high-density lipoprotein
HIC	hepatic iron content
HSC	hepatic stellate cells
HSFx	high-stability fucoxanthin
IRS-1	insulin receptor substrate 1
LCAT	lecithin cholesterol acyltransferase
LDL	low-density lipoprotein
MDA	malondialdehyde
MEK1	mitogen-activated protein kinase-1
MGIG	magnesium isoglycyrrhizinate
MT	metallothioneins
NAD	nicotinamide adenine dinucleotide
NADPH	nicotinamide adenine dinucleotide phosphate
NAFLD	on-alcoholic fatty liver disease
NAS	NAFLD Activity Score
NASH	non-alcoholic steatohepatitis
NHANES	National Health and Nutrition Examination Survey
OA	oleic acid
OS	oxidative stress
PNPLA3	patatin-like phospholipase domain-containing protein 3
PPARα	proliferator-activated receptor alpha
PPARγ	peroxisome proliferator-activated receptor gamma
RA	retinoic acid
RES	reticuloendothelial system
ROS	reactive oxygen species
SREBP	sterol regulatory element-binding protein
SOD	superoxide dismutase
T2DM	type 2 diabetes
TAK1	TGF-activated kinase-1
TCA	tricarboxylic acid
UCP2	uncoupling protein 2
UPR	unfolded protein response
VA	Vitamin A
WD	Wilson’s disease
